# Supplementary material for: Organic resolution function and effects of platinum nanoparticles on bacteria and organic matter
Source: PLoS One. 2019 Sep 19;14(9):e0222634. doi: 10.1371/journal.pone.0222634 (PMC6752831; doi:10.1371/journal.pone.0222634)

PLOS ONE

Organic resolution function and effects of non-colloidal platinum nanoparticles on bacteria and organic matter

--Manuscript Draft--

**Manuscript Number:**	PONE-D-19-16374
**Article Type:**	Research Article
**Full Title:**	Organic resolution function and effects of non-colloidal platinum nanoparticles on bacteria and organic matter
**Short Title:**	Organic resolution function of non-colloidal platinum nanoparticles
**Corresponding Author:**	Gomi Kazuhiro, Ph.D.<br>Tsurumi University, School of Dental Medicine<br>Yokohama, Kanagawa JAPAN
**Keywords:**	lipopolysaccharide; antibacterial effect; albumin; endotoxin
**Abstract:**	Rapid progress has been made in terms of metal nanoparticles studied in numerous fields. Metal nanoparticles have also been used in medical research, and antibacterial properties and anticancer effects have been reported. However, the underlying mechanism responsible for these effects has not been fully elucidated. Therefore, the present study focused on non-colloidal platinum nanoparticles (NC-PtNPs) and examined their antibacterial properties and functional potential for decomposing organic matter, considering potential applications in the dental field. NC-PtNPs were allowed to react with dental-related bacteria (Streptococcus mutans; Enterococcus faecalis, caries; Porphyromonas gingivalis, and endodontic and periodontal lesions). Antibacterial properties were evaluated by measuring colony formation. In addition, NC-PtNPs were allowed to react with albumin and lipopolysaccharides (LPSs), and the functional potential to decompose organic matter was evaluated. All evaluations were performed in vitro. Colony formation in all bacterial species was completely suppressed by NC-PtNPs at concentrations of >5 ppm. The addition of NC-PtNPs at concentrations of >10 ppm significantly increased fragmentation and decomposition. The addition of NC-PtNPs at concentrations of >125 pico/mL to 1 EU/mL LPS resulted in significant amounts of decomposition and elimination. The results revealed that NC-PtNPs had antibacterial effects against dental-related bacteria and proteolytic potential to decompose proteins and LPS, an inflammatory factor associated with periodontal disease. Therefore, the use and application of NC-PtNPs in periodontal and endodontic treatment is considered promising.
**Order of Authors:**	Hiroo Itohiya
	Yuji Matsushima
	Satoshi Shirakawa
	Sotarou Kajiyama
	Takatoshi Nagano
	Akihiro Yashima
	Gomi Kazuhiro, Ph.D.
**Additional Information:**	
**Question**	**Response**
**Financial Disclosure**<br><br>Enter a financial disclosure statement that describes the sources of funding for the work included in this submission. Review the submission guidelines for detailed requirements. View published research	The funders had no role in study design, data collection and analysis, decision to publish, or preparation of the manuscript.

Powered by Editorial Manager® and ProduXion Manager® from Aries Systems Corporation

articles from *PLOS ONE* for specific examples.<br><br>This statement is required for submission and **will appear in the published article** if the submission is accepted. Please make sure it is accurate.<br><br>**Unfunded studies**<br>Enter: *The author(s) received no specific funding for this work.*<br><br>**Funded studies**<br>Enter a statement with the following details:<br>• Initials of the authors who received each award<br>• Grant numbers awarded to each author<br>• The full name of each funder<br>• URL of each funder website<br>• Did the sponsors or funders play any role in the study design, data collection and analysis, decision to publish, or preparation of the manuscript?<br>• **NO** - Include this sentence at the end of your statement: *The funders had no role in study design, data collection and analysis, decision to publish, or preparation of the manuscript.*<br>• **YES** - Specify the role(s) played.<br><br>* typeset	
**Competing Interests**<br><br>Use the instructions below to enter a competing interest statement for this submission. On behalf of all authors, disclose any competing interests that could be perceived to bias this work—acknowledging all financial support and any other relevant financial or non-financial competing interests.<br><br>This statement **will appear in the published article** if the submission is accepted. Please make sure it is accurate. View published research articles from *PLOS ONE* for specific examples.	The authors have declared that no competing interests exist.

**NO authors have competing interests**<br><br>Enter: *The authors have declared that no competing interests exist.*<br><br>**Authors with competing interests**<br><br>Enter competing interest details beginning with this statement:<br><br>*I have read the journal's policy and the authors of this manuscript have the following competing interests: [insert competing interests here]*<br><br>* typeset	
**Ethics Statement**<br><br>Enter an ethics statement for this submission. This statement is required if the study involved:<br><br>• Human participants<br>• Human specimens or tissue<br>• Vertebrate animals or cephalopods<br>• Vertebrate embryos or tissues<br>• Field research<br><br>Write "N/A" if the submission does not require an ethics statement.<br><br>General guidance is provided below. Consult the submission guidelines for detailed instructions. **Make sure that all information entered here is included in the Methods section of the manuscript.**	N/A

Powered by Editorial Manager® and ProduXion Manager® from Aries Systems Corporation

**Format for specific study types**<br><br>**Human Subject Research (involving human participants and/or tissue)**<br>• Give the name of the institutional review board or ethics committee that approved the study<br>• Include the approval number and/or a statement indicating approval of this research<br>• Indicate the form of consent obtained (written/oral) or the reason that consent was not obtained (e.g. the data were analyzed anonymously)<br><br>**Animal Research (involving vertebrate animals, embryos or tissues)**<br>• Provide the name of the Institutional Animal Care and Use Committee (IACUC) or other relevant ethics board that reviewed the study protocol, and indicate whether they approved this research or granted a formal waiver of ethical approval<br>• Include an approval number if one was obtained<br>• If the study involved *non-human primates*, add *additional details* about animal welfare and steps taken to ameliorate suffering<br>• If anesthesia, euthanasia, or any kind of animal sacrifice is part of the study, include briefly which substances and/or methods were applied<br><br>**Field Research**<br><br>Include the following details if this study involves the collection of plant, animal, or other materials from a natural setting:<br>• Field permit number<br>• Name of the institution or relevant body that granted permission	
**Data Availability**<br><br>Authors are required to make all data underlying the findings described fully available, without restriction, and from the time of publication. PLOS allows rare exceptions to address legal and ethical concerns. See the PLOS Data Policy and FAQ for detailed information.	Yes - all data are fully available without restriction

A Data Availability Statement describing where the data can be found is required at submission. Your answers to this question constitute the Data Availability Statement and **will be published in the article**, if accepted.<br><br>**Important:** Stating 'data available on request from the author' is not sufficient. If your data are only available upon request, select 'No' for the first question and explain your exceptional situation in the text box.<br><br>Do the authors confirm that all data underlying the findings described in their manuscript are fully available without restriction?	
**Describe where the data may be found in full sentences. If you are copying our sample text, replace any instances of XXX with the appropriate details.**<br><br>• If the data are **held or will be held in a public repository**, include URLs, accession numbers or DOIs. If this information will only be available after acceptance, indicate this by ticking the box below. For example: *All XXX files are available from the XXX database (accession number(s) XXX, XXX.)*.<br>• If the data are all contained **within the manuscript and/or Supporting Information files**, enter the following: *All relevant data are within the manuscript and its Supporting Information files.*<br>• If neither of these applies but you are able to provide **details of access elsewhere**, with or without limitations, please do so. For example:<br><br>*Data cannot be shared publicly because of [XXX]. Data are available from the XXX Institutional Data Access / Ethics Committee (contact via XXX) for researchers who meet the criteria for access to confidential data.*<br><br>*The data underlying the results presented in the study are available from (include the name of the third party*	All relevant data are within the manuscript.

and contact information or URL).<br>• This text is appropriate if the data are owned by a third party and authors do not have permission to share the data.<br><br>* typeset	
Additional data availability information:	

Manuscript

Click here to download Manuscript Main_GMMOIJ-27_MI-1 KG.docx

Organic resolution function and effects of non-colloidal platinum nanoparticles on bacteria and organic matter

Short title: Organic resolution function of non-colloidal platinum nanoparticles

Hiroo Itohiya¶, Yuji Matsushima¶, Satoshi Shirakawa, Sotarou Kajiyama, Akihiro Yashima, Takatoshi Nagano, and Kazuhiro Gomi*

Department of Periodontology, Tsurumi University, School of Dental Medicine, 2-1-3 Tsurumi, Tsurumi ku, Yokohama, Japan 230-8501

*Corresponding author

E-mail: gomi-k@tsurumi-u.ac.jp (KG)

¶These authors contributed equally to this work.

Keywords: lipopolysaccharide, antibacterial effect, albumin, endotoxin

Abstract

Rapid progress has been made in terms of metal nanoparticles studied in numerous fields. Metal nanoparticles have also been used in medical research, and antibacterial properties and anticancer effects have been reported. However, the underlying mechanism responsible for these effects has not been fully elucidated. Therefore, the present study focused on non-colloidal platinum nanoparticles (NC-PtNPs) and examined their antibacterial properties and functional potential for decomposing organic matter, considering potential applications in the dental field. NC-PtNPs were allowed to react with dental-related bacteria (*Streptococcus mutans*; *Enterococcus faecalis,* caries; *Porphyromonas gingivalis,* and endodontic and periodontal lesions). Antibacterial properties were evaluated by measuring colony formation. In addition, NC-PtNPs were allowed to react with albumin and lipopolysaccharides (LPSs), and the functional potential to decompose organic matter was evaluated. All evaluations were performed *in vitro*. Colony formation in all bacterial species was completely suppressed by NC-PtNPs at concentrations of >5 ppm. The addition of NC-PtNPs at concentrations of >10 ppm significantly increased fragmentation and decomposition. The addition of NC-PtNPs at concentrations of >125 pico/mL to 1 EU/mL LPS resulted in significant amounts of decomposition and elimination. The results revealed that NC-PtNPs had antibacterial effects against dental-related bacteria and proteolytic potential to decompose proteins and LPS, an inflammatory

factor associated with periodontal disease. Therefore, the use and application of NC-PtNPs in periodontal and endodontic treatment is considered promising.

Introduction

Metal nanoparticles, which are obtained by converting metals into fine particles (< 100 nm in diameter), have a large surface area and exhibit properties that differ from those of bulk metals because of the quantum size effect [1]. Metal nanoparticles have been studied in numerous fields, such as chemistry, biology, materials science, and medicine, and rapid advances have been reported in recent years [2-6]. The antibacterial characteristics of metal nanoparticles (silver, zinc oxide, copper oxide, etc.) have previously been reported [7-12]. In particular, silver nanoparticles (AgNPs) have attracted much interest in many fields due to their excellent broad-spectrum antibacterial activity [9,13]. In contrast, gold nanoparticles (AuNPs) are commonly evaluated for use in biosensing or drug delivery applications, given that these particles are inert and highly stable [14]. Antibacterial AuNPs are produced by coating AuNPs with organic molecules that have antibacterial properties [15].

Previous studies have reported the antibacterial effects of platinum nanoparticles (PtNPs). For example, PtNPs slow cell division in *Escherichia coli* [16]. Furthermore, PtNPs, which are clusters of Pt atoms in the range of 1–100 nm, are attracting attention due to the extremely high

catalytic activity. Recently, the ability of these nanoparticles to suppress inflammation has also been evaluated [17,18]. Chemical interactions are thought to be induced by contact between bacteria and PtNPs, leading to the decomposition of bacteria. This is believed to trigger a chain reaction in which free radicals produced from the antioxidant response of PtNPs damage bacteria [19]. While one study has found that PtNPs increase the level of intracellular reactive oxygen species (ROS) [20], another study has reported that PtNPs eliminate ROS [21].

In the present study, we focused on the application of metal nanoparticles in the dental field because platinum is a precious metal that is unlikely to cause allergies, does not demonstrate genotoxic potential, and has high potential for clinical application [15].

Although metal nanoparticles possess various useful properties, nanoparticles clump into a large aggregate when left in their original state, altering their properties. To maintain the stable dispersed state of nanoparticles, protective agents are generally added to adsorb and configure on the surface of metal nanoparticles and to prevent nanoparticle aggregation [22]. However, the use of protective agents reportedly hinders expression of the full range of properties that metal nanoparticles originally possess [23].

Because PtNPs are not protected by organic matter (colloidal film) and metal particles exist in the intact state, the use of non-colloidal platinum nanoparticles (NC-PtNPs) may be more effective.

Indeed, by not using protective materials, such as organic matter, on metal particles, the activity of metal nanoparticles remains high. With the use of metal nanoparticles, antibacterial properties and antioxidant effects are expected to be stronger than those with conventional metal colloids. In addition, as platinum particles do not dissolve in water, they do not ionize; therefore, long-term activity can be expected. The aim of this study was to investigate the functional potential of nanoparticles (NC-PtNPs) to decompose organic matter and antibacterial activity against dental-related bacteria, which are important indicators of the potential application of NC-PtNPs in the field of dentistry.

Materials and methods

In this experiment, NC-PtNPs (particle size: 2–19 nm) were obtained from the manufacturer (Ceramics Craft Co. Ltd. Shizuoka, Japan). NC-PtNPs were suspended in sterile, purified water before use.

Measurement of antibacterial activity

Bacterial culture and regulation

Experiments were performed with bacteria that are important in dentistry, namely the *Streptococcus mutans* ATCC25175 strain, *Enterococcus faecalis* ATCC19433 strain, and *Porphyromonas gingivalis* ATCC 33277 strain. *Streptococcus mutans* is a gram-positive bacterium

that causes caries; *E. faecalis* is a gram-positive bacterium that causes refractory apical periodontitis; and *P. gingivalis* is a gram-negative obligate anaerobic and a periodontal pathogenic bacterium. All bacteria were purchased from American Type Culture Collection (ATCC), VA, USA. *Streptococcus mutans* was cultured in Tryptic Soy Broth(TSB, FUJIFILM Wako Pure Chemical Co., Osaka, Japan) and agar medium (Becton Dickinson) with 4% glucose (FUJIFILM Wako Pure Chemical Co.) at 37 °C under aerobic conditions for 2 days, followed by preculture in TSB liquid medium with 4% glucose under aerobic shaking for 2 days. *Enterococcus faecalis* was cultured in TSB agar medium (at 37 °C under aerobic conditions for 2 days), followed by preculture in TSB liquid medium under aerobic shaking for 2 days. *Porphyromonas gingivalis* was anaerobically cultured in an anaerobic culturing device (ANX-1, Hirasawa, Tokyo, Japan) under N_2: 80%, CO_2: 10%, and H_2: 10% conditions at 37 °C for 1 week.

Precultured *S. mutans*, *E. faecalis*, and *P. gingivalis* were centrifuged for 5 minutes at 4 °C and 7,000 ×g, harvested, and suspended in distilled water. Bacterial counts were adjusted to 10^5–10^6 CFU/mL at an optical density of 620 nm with a spectrophotometer (UV-1200, SHIMAZU Co., Kyoto, Japan).

Antibacterial testing

NC-PtNPs were added to 100 µl of 1×10^4 CFU/mL bacterial solutions to achieve final concentrations of 1, 5, 10, and 20 ppm. Samples that had been allowed to react for 10 minutes were inoculated onto bacterial culture medium. The associated antibacterial effects were determined based on the presence or absence of colony formation on the medium. The group without NC-PtNPs in the bacterial medium was defined as the positive control group, and the group to which lipopolysaccharides (LPSs) were not added was defined as the negative control group.

Measurement of functional potential for organic resolution

Electrophoresis and BCA protein assay

To investigate the functional potential for organic resolution of NC-PtNP solutions, NC-PtNP solutions were added to albumin suspensions and were allowed to react (experimental groups), and the state of decomposition was observed. NC-PtNP solutions (100 ppm) were added to 2 mg/mL albumin aqueous solution to achieve a final concentration of 10 ppm. The mixture was stirred and allowed to react at room temperature for 1, 10, 30, and 60 minutes. In addition, suspension with only albumin was defined as the control group. Functional potential for organic resolution was evaluated through protein abundance measurement using polyacrylamide electrophoresis and the bicinchoninic acid (BCA) assay.

Electrophoresis assay

Samples collected from the experimental and control groups were electrophoresed using 15% polyacrylamide containing 1% sodium dodecyl sulfate (SDS) according to the Laemmli method [24]. In other words, each sample was dissolved in 1 mL of 0.01 mol/l Tris-HCL buffer solution (pH 6.8) containing 1% SDS and 25% glycerin to be used as samples for electrophoresis. After electrophoresis at 40 mA for 80 minutes under a low-voltage condition using the electrophoresis tank (Pagel AE-600, SPS-15S, ATTO Co., Japan), staining was performed with Coomassie brilliant blue (CBB, Bio-Rad, USA), followed by decoloration with stain/destain solution (Bio-Rad), and bands were detected.

BCA assay

The amount of albumin in each collected sample was measured using the BCA assay [25]. Overall, 200 µl of each sample was dispensed to a 96-well multiplate, allowed to react for 15 minutes according to the manual of the protein assay kit (Pierce Protein assay kit, Thermo SCIENTIFIC), and absorbance was measured (E-MAX Plus Microplate reader, Molecular Devices Co., Japan) at 562 nm.

In this bacterial culture assay, the only standard was a sample with no bacterial growth at a concentration of 10 ppm.

Measurement of ability to decompose lipopolysaccharides

We studied whether LPS, a pathogenic factor secreted by some periodontal bacteria, could be decomposed by NC-PtNPs. NC-PtNPs were allowed to react with LPS, and the amount of LPS was measured. The Toxin Sensor Chromogenic LAL Endotoxin Assay Kit (Gen Script Co., USA) was used in the measurement of LPS. NC-PtNP solutions at 0, 10, or 50 ppm were added to 0.625, 1.25, 2.5, 5.0, and 10.0 EU/mL LPS. The mixture was allowed to react in a tube placed at room temperature for 10 minutes. The amount of LPS was measured by measuring the absorbance at 545 nm. Furthermore, to investigate the effective concentration of NC-PtNPs against 1 EU/mL of LPS, a serially diluted NC-PtNP solution (0.015625–1 ppm) was added. The mixture components were then allowed to react for 10 minutes, at which point absorbance was measured, and LPS was detected.

Statistical analysis

In the colorimetric determination of albumin and LPS decomposition ability, obtained values were subjected to one-way analysis of variance (one-way ANOVA) and Tukey’s multiple

comparison tests, with significance levels for both set at 0.05. All statistical analyses were performed using SPSS Statistics Version 11.5 (IBM Japan).

Results

Antibacterial activity of NC-PtNP solutions

To investigate the antibacterial activity of NC-PtNPs, NC-PtNPs were added to *S. mutans*, *E. faecalis*, and *P. gingivalis* bacterial solutions to achieve concentrations of 1, 5, 10, and 20 ppm. Samples that had been allowed to react for 10 minutes were inoculated onto each medium, and the presence or absence of colonies was confirmed to determine the antibacterial effect. Colony formation was completely suppressed in all bacterial strains at concentrations of >5 ppm. However, at a concentration of 1 ppm, colonies exhibited growth equivalent to that in the negative control group (which had not been treated with NC-PtNPs). The same trend was observed for all bacterial strains investigated in this study (Fig 1).

Fig 1. Antibacterial activity of non-colloidal nanoplatinum. Non-colloidal nanoplatinum aqueous solutions (concentrations of 1, 5, 10, and 20 ppm) were added to bacterial solutions of 1×10^4 *Streptococcus mutans*, *Enterococcus faecalis*, *or Porphyromonas gingivalis* and then allowed to react

for 10 minutes. At concentrations up to 5 ppm, colony formation was completely suppressed in all bacterial strains.

Functional potential for organic resolution of NC-PtNPs

Albumin decomposition

To investigate the functional potential of NC-PtNPs for organic resolution, albumin suspensions were allowed to react with NC-PtNPs. The results were visualized using polyacrylamide electrophoresis. A comparison between the bands of control albumin and albumin that had been exposed to NC-PtNPs in the electrophoretic image revealed a time-dependent decrease in the bandwidth of albumin in the experimental groups (Fig 2). In addition, smear layers believed to be albumin decomposition products were observed beneath the bands.

Fig 2. Functional potential of non-colloidal nanoplatinum solutions to decompose albumin. Overall, 10 ppm non-colloidal nanoplatinum solutions were added to 2 mg/mL albumin suspension, and the state of samples, which had been allowed to react for 1, 10, 30, and 60 minutes, was confirmed using electrophoresis. A time-dependent decrease in bandwidth was observed, and smear

layers believed to be albumin decomposition products were observed beneath the bands. (M.W.: molecular weight)

The measurement of protein abundance using the BCA protein assay revealed findings in line with those observed in the electrophoretic image. When compared with the absorbance in the control group, the absorbance of the experimental groups, which had been exposed to NC-PtNPs, decreased significantly ($p < 0.05$) after 1 minute of activity and became almost constant after 30 minutes (Fig 3).

Fig 3. Functional potential of non-colloidal nanoplatinum solutions to decompose albumin. Overall, 10 ppm non-colloidal nanoplatinum solutions were added to 2 mg/mL albumin solutions and allowed to react for 1, 10, 30, and 60 minutes. Protein abundance was measured in the BCA protein assay. (*$p < 0.05$, Tukey's test)

LPS decomposition

To study LPS decomposition, NC-PtNPs (10 and 50 ppm) were allowed to react with LPS adjusted to 10.0, 5.0, 2.5, 1.25, or 0.625 EU/mL for 10 minutes. At 10 minutes, the amount of LPS

was measured. The results showed that absorbance decreased significantly with the addition of NC-PtNPs in all groups (Fig 4). Next, to study the minimum effective concentration of NC-PtNPs, serially diluted solutions of 1 ppm NC-PtNPs were added to 1 EU/mL LPS. The mixture was allowed to react for 10 minutes. The results showed that LPS was significantly degraded ($p < 0.05$) compared with the control at a concentration of 0.125 ppm NC-PtNPs. At concentrations of >0.25 ppm, LPS values equivalent to those of the negative control group, which had not been exposed to LPS, were confirmed (Fig 5).

Fig 4. Functional potential of non-colloidal nanoplatinum solutions to decompose LPS.

Overall, 10 and 50 ppm NC-PtNPs were added to LPS at different concentrations and allowed to react for 10 minutes. LPS decreased in all groups. (* $p < 0.05$, Tukey's test)

Fig 5. Effective concentration of non-colloidal nanoplatinum solution against LPS.

NC-PtNPs at different concentrations were added to 1 EU/mL LPS and allowed to react for 10 minutes. At concentrations of >0.25 ppm, LPS was decomposed to a level equivalent to that in the negative control group, where no LPS had been added. In the positive control groups, LPS without non-colloidal nanoplatinum solutions was added. (*$p < 0.05$, Tukey's test)

Discussion

Aqueous solutions of nano-sized metal particles reportedly exhibit more potent antibacterial activity as particle size enters the nanometer range [26]. In particular, AgNPs are known to have excellent antibacterial effects, but they are also known to possess strong cytotoxicity [27]. Recently, metal NPs have attracted much attention not only due to their antibacterial properties but also because of their application in other areas of medicine, such as cancer treatment [28]. This study focused on platinum, a stable metal that does not tend to induce allergic reactions, and investigated the effects of platinum nanoparticles. The surfaces of colloidal PtNPs are typically coated with organic matter to prevent the aggregation of NPs [29]. However, this study was performed using NC-PtNPs, which have uncoated particle surfaces. When NC-PtNP solutions were added to *S. mutans*, *E. faecalis*, and *P. gingivalis* adjusted to 1×10^4 cells/mL, clear antibacterial properties were observed at concentrations of > 5 ppm. In a similar experiment that was previously reported, nanoplatinum particles (2–19 nm) were allowed to react for 15 minutes. These nanoparticles exhibited the same levels of antibacterial activity as that reported in our study, even though the bacterial strains were different [30,31]. The reaction time of the solution used and platinum particle size were almost the same, and the results obtained were similar to those reported in our study. Kanieczny et al. conducted

a study to compare the antibacterial properties against gram-positive and gram-negative bacteria using PtNPs. The results showed a stronger antibacterial effect against gram-negative bacteria [32] because of the thin cell wall of gram-negative bacteria. In addition, Rosenberg et al. have also reported on antibacterial activity against *E. coli*, a gram-negative bacterium [16]. In this study, PtNPs demonstrated strong antibacterial activity not only against *P. gingivalis*, a gram-negative bacterium, but also against *S. mutans* and *E. faecalis*, which are gram-positive bacte[illegible] Unlike previous studies, this study was performed with NC-PtNPs, in which the NP surface was not coated with organic matter, which is believed to be the reason for the differing results observed.

A possible mechanism for antibacterial effects includes damage to the cell wall or cell membrane, leading to the outflow of cytoplasm and invasion of NPs into the cell. NPs bind with proteins, stopping cell function and ultimately killing the bacterium [10]. Through transmission electron microscopic images, Chwalibog et al. have shown that PtNP-mediated damage to bacterial cell walls allows cytoplasm to flow out of the cell [33,34]. However, the mechanism by which metal NPs damage cell walls and invade the bacterium has not been fully elucidated. However, if cell walls and cytoplasm, which comprise proteins, are damaged, PtNPs may exhibit proteolytic effects. We therefore investigated whether PtNPs possessed functional potential to decompose organic matter.

The results showed a time-dependent decrease in the bandwidth of albumin in the group

where NC-PtNPs (10 ppm) had been exposed to albumin (2 mg/mL) compared with experiments in which albumin was electrophoresed alone. Although albumin may have been adsorbed to PtNPs, a smear layer believed to be the decomposition products of albumin appeared beneath the band after 1 minute of reaction time. The bandwidth of albumin decreased as the reaction time increased, and the smear layers underneath gradually disappeared. Albumin was therefore considered to have decomposed in a time-dependent manner. In addition, protein abundance was measured using the BCA protein assay with the same samples. The results obtained up to the 30-min timepoint showed a significant time-dependent decrease in the amount of proteins in the experimental groups compared with the control group; protein levels did not decrease after 30 minutes. The decrease in bandwidth in the electrophoretic image, as well as the time-dependent decrease in the amount of protein revealed by the results of the BCA protein assay, indicates the decomposition of albumin by NC-PtNPs.

Next, we studied the effects of NC-PtNP solution on LPS, a constituent of gram-negative bacterial cell walls. PtNPs were added to 0.625–10.0 EU LPS to achieve final concentrations of 10 and 50 ppm. The mixture was allowed to react for 10 minutes. The results showed that LPS decreased significantly in all experimental groups compared with the control group. This might suggest a strong ability of PtNPs to decompose LPS. Next, to detect the minimum effective concentration of PtNPs required to decompose 1 EU/mL LPS, NC-PtNPs at concentrations of 0.01561–1 ppm were allowed

to react for 10 minutes. The amount of LPS remaining was then measured. PtNP concentrations of up to 0.25 ppm showed almost the same values as those of the negative control group, suggesting LPS decomposition. Conversely, at concentrations of ≤0.0625 ppm, no significant difference was noted in the positive control group.

Periodontal diseases, which are prevalent worldwide, stem from infection with periodontal pathogens, which are typically gram-negative bacteria [35]. In addition, even after the periodontal pathogens die, LPS, a bacterial constituent, remains on the root surface, acts as an endotoxin, and causes inflammation in periodontal tissues [36]. The NC-PtNPs used in this study were of a particle size of 2–19 nm and were non-colloidal particles with exposed metal surfaces. These NPs can decompose organic matter (especially LPS) effectively, even when used at extremely low concentrations. Therefore, NC-PtNPs may be an effective tool for periodontal treatment by acting as antibacterial agents against periodontal pathogenic bacteria. NC-PtNPs may be used to eliminate LPS that remains on the root surface, rendering the root surface innocuous to periodontal tissue. In addition, during endodontic treatment, it is difficult to kill bacteria that have entered the dentinal tubules. However, by using NC-PtNPs in root canals, nanoparticles may enter the dentinal tubules and kill the bacteria. However, this study was only a limited basic scientific research effort, and further studies are necessary to determine the full potential of such nanoparticles for clinical applications.

Conclusion

NC-PtNPs have been shown to mediate antibacterial effects related to caries, endodontic lesions, and periodontal diseases. NC-PtNPs also exhibit functional potential to decompose proteins and strong effectiveness against LPS, the cell wall constituent of gram-negative bacteria. However, this study was limited to only *in vitro* findings, and there is a necessity to further investigate whether similar results can be obtained in the clinic.

Acknowledgments

The authors are grateful to Dr. Takuma Suzuki for assistance in measuring the amount of LPS. We thank Crimson Interactive Pvt. Ltd. (Ulatus) – www.ulatus.jp for their assistance in manuscript translation and editing.

References

1. Roduner E. Size matters: why nanomaterials are different. Chem Soc Rev. 2006;35: 583-592. doi: 10.1039/b502142c.
2. Schmid G. Clusters and Colloids from Theory to Application VCH, Weinheim; 1994.
3. Corain B, Schmid G, Toshima N, editors. Metal Nano-cluster in Catalysis and Materials Science: The Issue of Size control. Elsevier, Amsterdam; 2008.
4. Silvert PY, Herrera-Urbina R, Tekaia-Elhsissen K. Preparation of colloidal silver dispersions by the polyol process. Part 1—Synthesis and characterization. J Mater Chem. 1996;6: 573-577. doi: 10.1039/JM9960600573.
5. Toshima N, Yonezawa T. Bimetallic nanoparticles - materials for chemical and physical applications. New J Chem. 1998;22: 1179-1201. doi: 10.1039/a805753b.
6. Wieckowski A, Savinova ER, Vayenas CG, editors. Catalysis and electrocatalysis at nanoparticle surfaces. CRC Press; 2003.
7. Taylor E, Webster TJ. Reducing infections through nanotechnology and nanoparticles. Int J

Nanomedicine. 2011;6: 1463. doi: 10.2147/IJN.S22021.

8. Veerapandian M, Yun K. Functionalization of biomolecules on nanoparticles specialized for antibacterial applications. Appl Microbiol Biotechnol. 2011;90: 1655-1667. doi: 10.1007/s00253-011-3291-6.

9. Yamada M, Foote M, Prow TW. Therapeutic gold, silver, and platinum nanoparticles. WIREs Nanomed Nanobiotechnol. 2015;7: 428-445. doi: 10.1002/wnan.1322.

10. Wang L, Hu C, Shao L. The antimicrobial activity of nanoparticles: present situation and prospects for the future. Int J Nanomed. 2017;14: 1227-1249. doi: 10.2147/IJN.S121956.

11. McGuffie MJ, Hong J, Bahng JH, Glynos E, Green PF, Kotov NA, et al. Zinc oxide nanoparticle suspensions and layer-by-layer coatings inhibit staphylococcal growth. Nanomedicine. 2016;12: 33-42. doi: 10.1016/j.nano.2015.10.002.

12. Su Y, Zheng X, Chen Y, Li M, Liu K. Alteration of intracellular protein expressions as a key mechanism of the deterioration of bacterial denitrification caused by copper oxide nanoparticles. Sci Rep. 2015;5: 15824. doi: 10.1038/srep15824.

13. Rai M, Yadav A, Gade A. Silver nanoparticles as a new generation of antimicrobials. Biotechnol Adv. 2009;27: 76-83. doi: 10.1016/j.biotechadv.2008.09.002.

14. Rai A, Prabhune A, Perry CC. Antibiotic mediated synthesis of gold nanoparticles with potent

antimicrobial activity and their application in antimicrobial coatings. J Mater Chem. 2010;20: 6789-6798. doi: 10.1039/c0jm00817f.

15. Yang X, Yang J, Wang L, Ran B, Jia Y, Zhang L, et al. Pharmaceutical Intermediate-modified gold nanoparticles: Against multidrug-resistant bacteria and wound-healing application via an electrospun scaffold. ACS Nano. 2017;11: 5737-5745. doi: 10.1021/acsnano.7b01240.

16. Rosenberg B, Vancamp L, Krigas T. Inhibition of cell division in Escherichia coli by electrolysis products from a platinum electrode. Nature. 1965;205: 698-699. doi: 10.1038/205698a0.

17. Sawosz E, Chwalibog A, Szeliga J, Sawosz F, Grodzik M, Rupiewicz M, et al. Visualization of gold and platinum nanoparticles interacting with Salmonella enteritidis and Listeria monocytogenes. Int J Nanomedicine. 2010;5: 631-637. doi: 10.2147/IJN.S12361.

18. Chwalibog A, Sawosz E, Hotowy A, Szeliga J, Mitura S, Mitura K, et al. Visualization of interaction between inorganic nanoparticles and bacteria or fungi. Int J Nanomedicine. 2010;5: 1085-1094. doi: 10.2147/IJN.S13532.

19. Konieczny P, Goralczyk AG, Szmyd R, Skalniak L, Koziel J, Filon FL, et al. Effects triggered by platinum nanoparticles on primary keratinocytes. Int J Nanomedicine. 2013;8: 3963-3975. doi: 10.2147/IJN.S49612.

20. Horie M, Kato H, Endoh S, Fujita K, Komaba LK, Nishio K, et al. Cellular effects of industrial

metal nanoparticles and hydrophilic carbon black dispersion. J Toxicol Sci. 2014;39: 897-907. doi: 10.2131/jts.39.897.

21. Onizawa S, Aoshiba K, Kajita M, Miyamoto Y, Nagai A. Platinum nanoparticle antioxidants inhibit pulmonary inflammation in mice exposed to cigarette smoke. Pulm Pharmacol Ther. 2009;22: 340-349. doi: 10.1016/j.pupt.2008.12.015.

22. Mayer AB. Colloidal metal nanoparticles dispersed in amphiphilic polymers. Polym Adv Technol. 2001;12:96-106. doi: 10.1002/1099-1581(200101/02)12:1/2<96::AID-PAT943>3.0.CO;2-G.

23. Ma S, Izutani N, Imazato S, Chen JH, Kiba W, Yoshikawa R, et al. Assessment of bactericidal effects of quaternary ammonium-based antibacterial monomers in combination with colloidal platinum nanoparticles. Dent Mater J. 2012;31: 150-156. doi: 10.4012/dmj.2011-180.

24. Laemmli UK. Cleavage of structural Proteins during the assembly of the Head of Bacteriophage T4. Nature. 1970;227: 680-685. doi: 10.1038/227680a0.

25. Brown RE, Jarvis KL, Hyland KJ. Protein measurement using bicinchoninic acid: elimination of interfering substances. Anal Biochem. 1989;180: 136-139. doi: 10.1016/0003-2697(89)90101-2.

26. Gopal J, Hasan N, Manikandan M, Wu HF. Bacterial toxicity/compatibility of platinum nanospheres, nanocuboids and nanoflowers. Sci Rep. 2013;3: 1260. doi: 10.1038/srep01260.

27. Mao BH, Tsai JC, Chen CW, Yan SJ, Wang YJ. Mechanisms of silver nanoparticle-induced

toxicity and important role of autophagy. Nanotoxicology. 2016;10: 1021-1040. doi: 10.1080/17435390.2016.1189614.

28. Yhee JY, Son S, Lee H, Kim K. Nanoparticle-based combination therapy for cancer treatment. Curr Pharm Des. 2015;21: 3158-3166. doi: 10.2174/1381612821666150531165059.

29. Lin CS, Khan MR, Lin SD. The preparation of Pt nanoparticles by methanol and citrate. J Colloid Interface Sci. 2006;299: 678-685. doi: 10.2174/1381612821666150531165059.

30. Chwalibog A, Sawosz E, Hotowy A, Szeliga J, Mitura S, Mitura K, et al. Visualization of interaction between inorganic nanoparticles and bacteria or fungi. Int J Nanomedicine. 2010;5: 1085-1094. doi: 10.2147/IJN.S13532.

31. Sawosz E, Chwalibog A, Szeliga J, Sawosz F, Grodzik M, Rupiewicz M, et al. Visualization of gold and platinum nanoparticles interacting with Salmonella enteritidis and Listeria monocytogenes. Int J Nanomedicine. 2010;5: 631-637. doi: 10.2147/IJN.S12361.

32. Konieczny P, Goralczyk AG, Szmyd R, Skalniak L, Koziel J, Filon FL, et al. Effects triggered by platinum nanoparticles on primary keratinocytes. Int J Nanomedicine. 2013;8: 3963-3975. doi: 10.2147/IJN.S49612.

33. Chwalibog A, Sawosz E, Hotowy A, Szeliga J, Mitura S, Mitura K, et al. Visualization of interaction between inorganic nanoparticles and bacteria or fungi. Int J Nanomedicine. 2010;5:

1085-1094. doi: 10.2147/IJN.S13532.

34. Sawosz E, Chwalibog A, Szeliga J, Sawosz F, Grodzik M, Rupiewicz M, et al. Visualization of gold and platinum nanoparticles interacting with Salmonella enteritidis and Listeria monocytogenes. Int J Nanomedicine. 2010;5: 631-637. doi: 10.2147/IJN.S12361.

35. Socransky SS, Haffajee AD. The bacterial etiology of destructive periodontal disease: current concepts. J Periodontol. 1992;63: 322-331. doi: 10.1902/jop.1992.63.4s.322.

36. Hanazawa S, Nakada K, Ohmori Y, Miyoshi T, Amano S, Kitano S. Functional role of interleukin 1 in periodontal disease: induction of interleukin 1 production by Bacteroides gingivalis lipopolysaccharide in peritoneal macrophages from C3H/HeN and C3H/HeJ mice. Infect Immun. 1985;50: 262-270.

Figure 1 Click here to download Figure Fig 1_GMMOIJ-28.tif

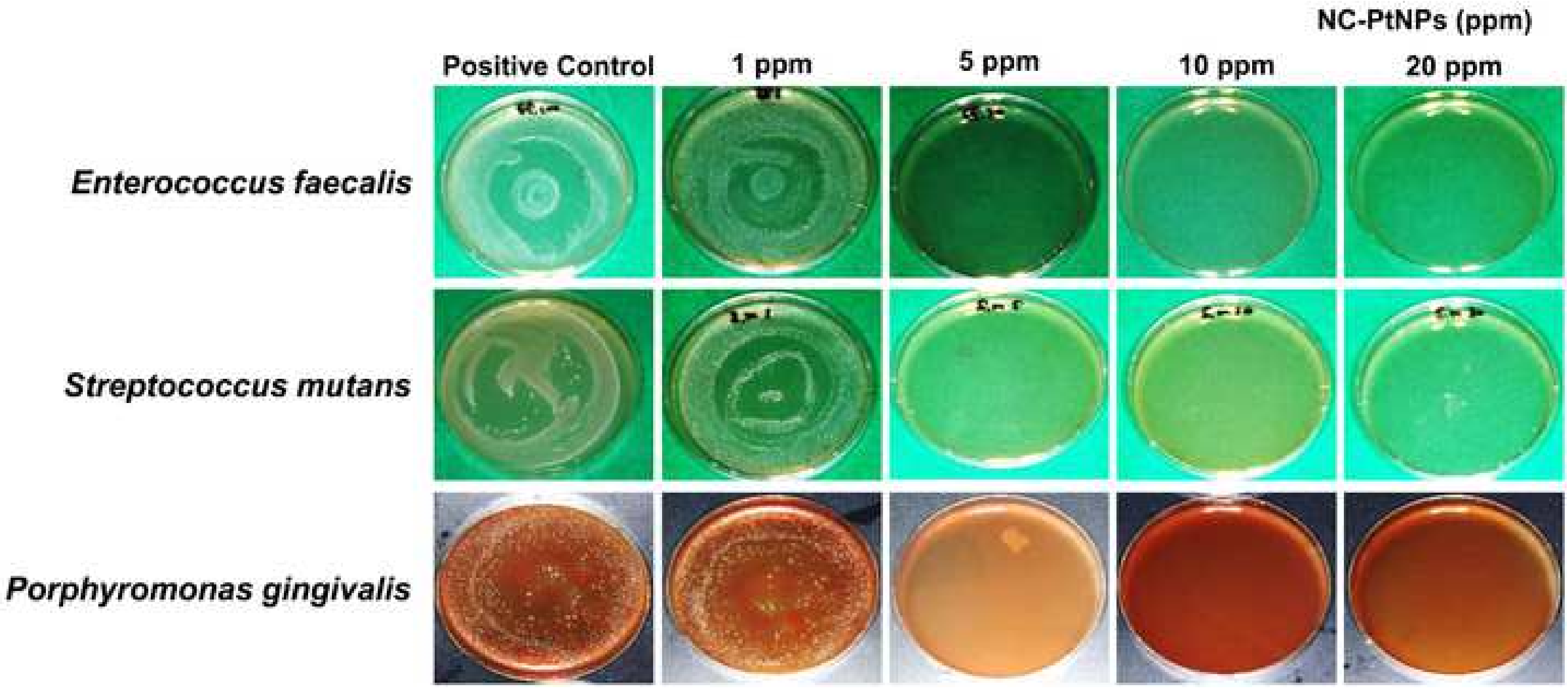

Figure 2

Click here to download Figure Fig 2_GMMOIJ-28.tif

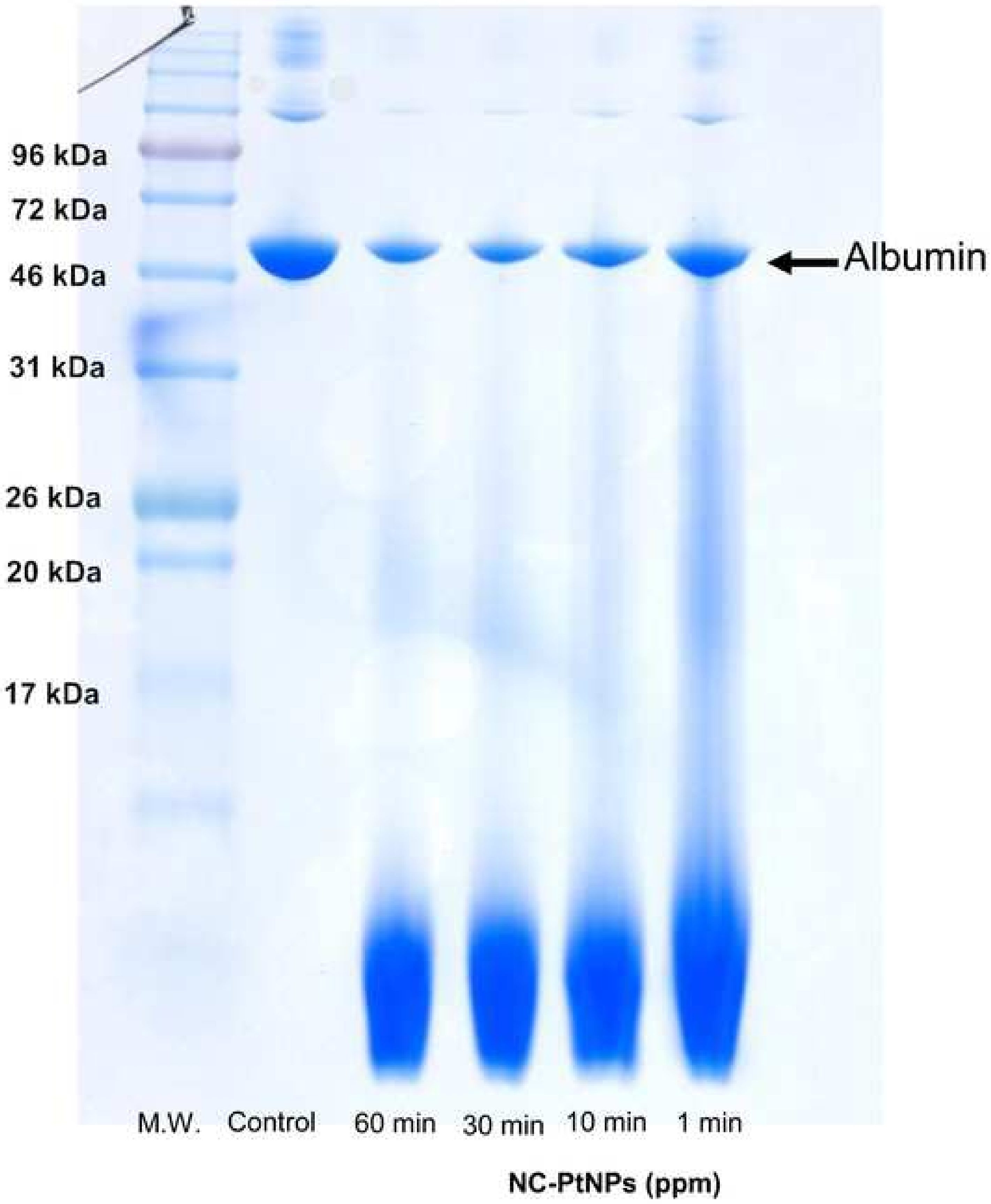

Figure 3

Click here to download Figure Fig 3_GMMOIJ-28.tif

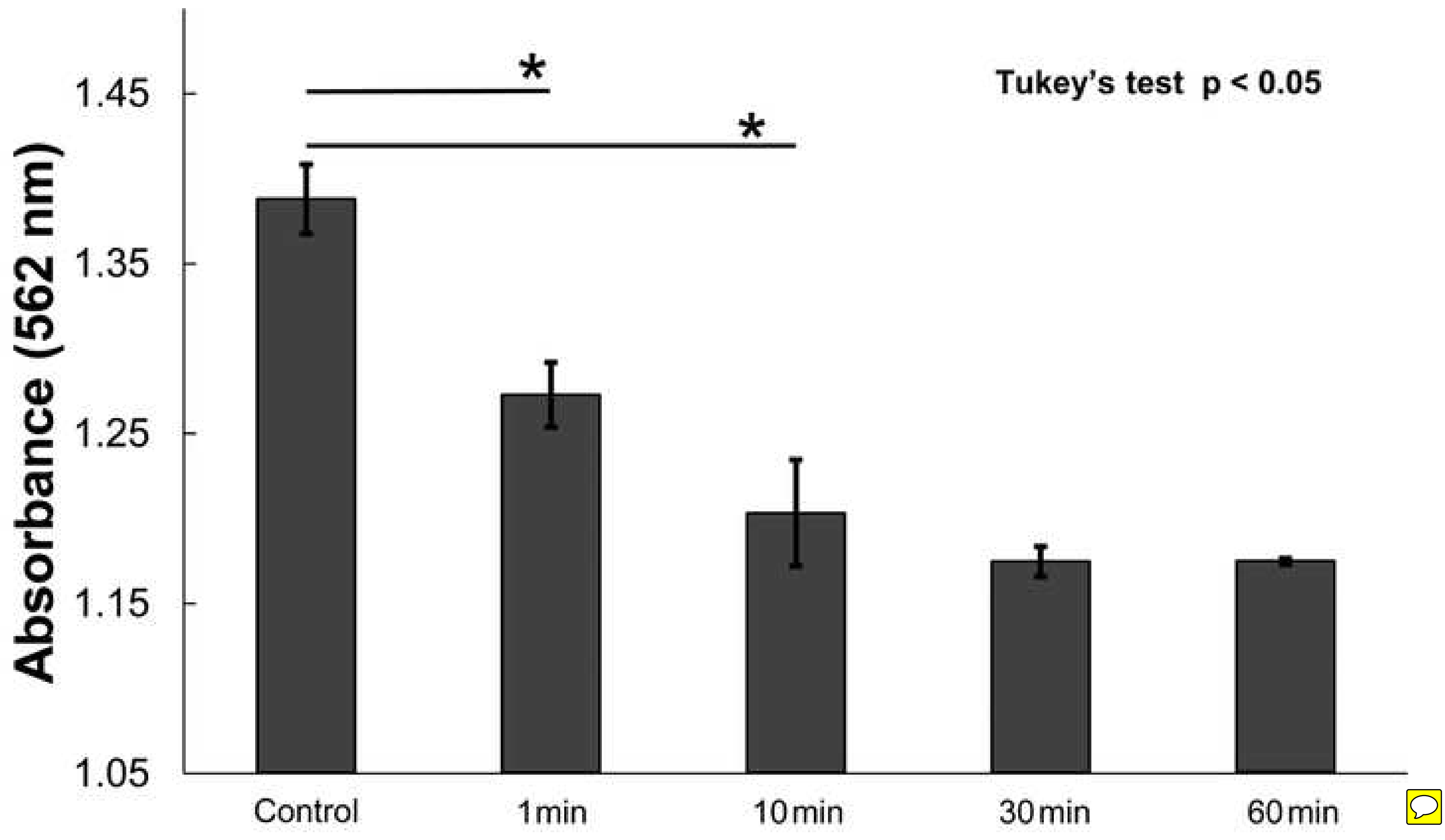

Figure 4

Click here to download Figure Fig 4_GMMOIJ-28.tif

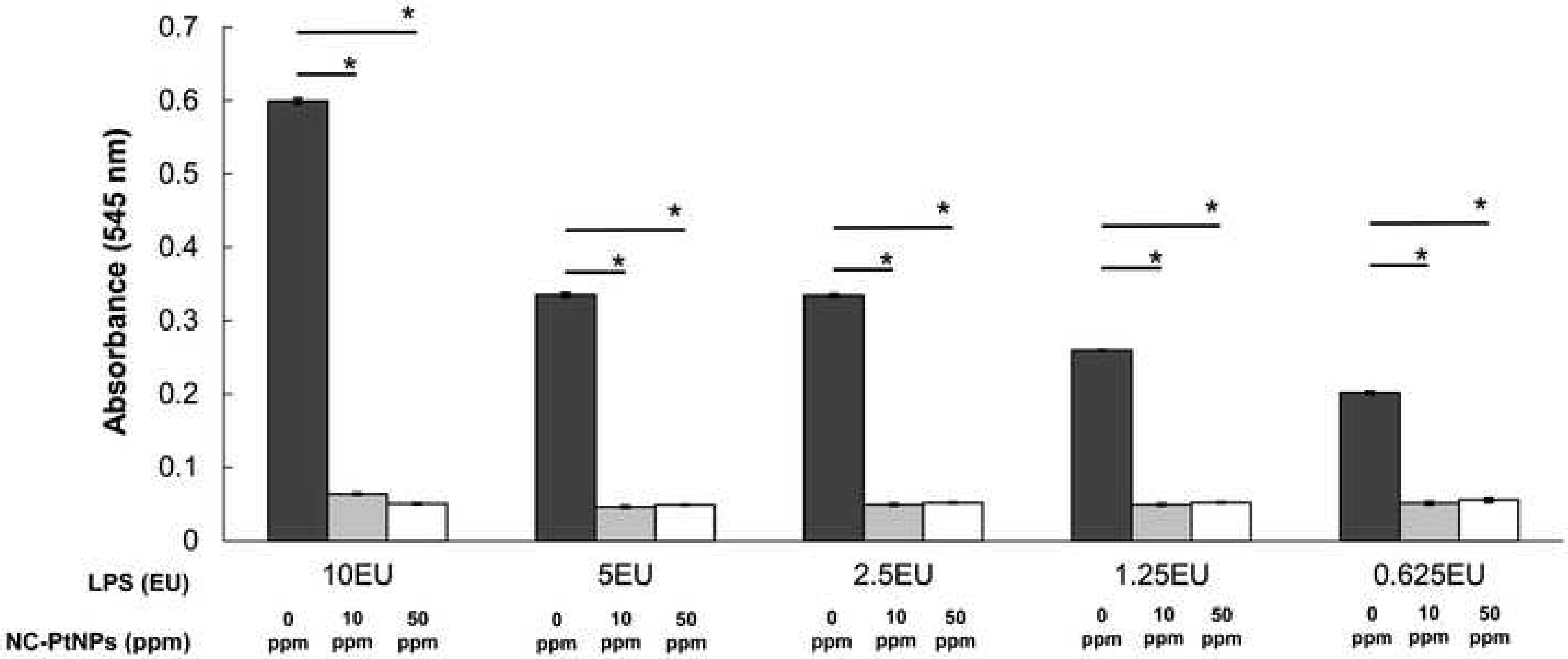

Figure 5

Click here to download Figure Fig 5_GMMOIJ-28.tif

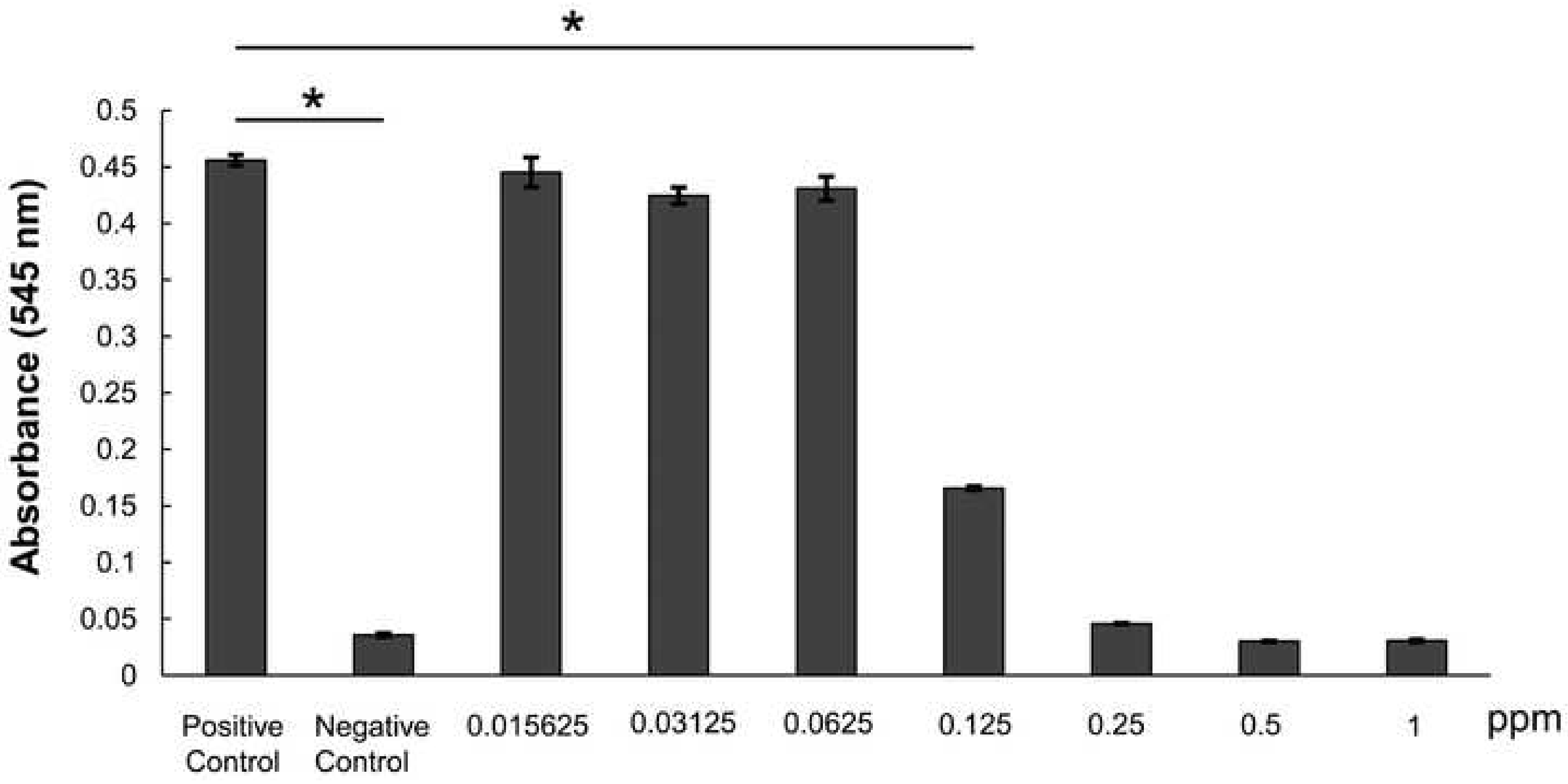

Supplement: Attachment — Submitted filename: PONE-D-19-16374_reviewer comments 1.pdf [file pone.0222634.s004.pdf]
