# Supplementary material for: Organic resolution function and effects of platinum nanoparticles on bacteria and organic matter
Source: PLoS One. 2019 Sep 19;14(9):e0222634. doi: 10.1371/journal.pone.0222634 (PMC6752831; doi:10.1371/journal.pone.0222634)
Supplement: Attachment — Submitted filename: Response to Reviewers.docx [file pone.0222634.s005.docx]

Dear Reviewers,

We would like to thank you and the reviewers for the valuable comments on the original version of our manuscript. We have taken all these comments into account and submit, herewith, a revised version of our paper. We have addressed all the comments by reviewers, as indicated in the attached pages, and we hope that the explanations are satisfactory.

We hope that the revised version of our paper is now suitable for publication in the PLOS ONE, and we look forward to hearing from you at the earliest.

Sincerely Yours,

Kazuhiro Gomi

Reviewer 1

1. Q: In material method section (Bacterial culture and regulation), write down the parameters for shaking at what rpm shaking was kept for aerobic bacteria.

→ A: I changed the description as follows according to your indication.

P6, line 96, 99 as follow.

Streptococcus mutans was cultured in Tryptic Soy Broth (TSB, FUJIFILM Wako Pure Chemical Co., Osaka, Japan) and agar medium (Becton Dickinson) with 4% glucose (FUJIFILM Wako Pure Chemical Co.) at 37 ℃ under aerobic conditions for 2 days, followed by preculture in TSB liquid medium with 4% glucose under aerobic shaking at 100 rpm in shaking incubator (SI-782, Optima Inv. Tokyo, Japan) for 2 days. Enterococcus faecalis was cultured in TSB agar medium (at 37 ℃ under aerobic conditions for 2 days), followed by preculture in TSB liquid medium under aerobic shaking at 100 rpm in shaking incubator for 2 days.

2. Q: Add a section of stock solution preparation of NPs under material method.

→ A: We added the information about preparation of PtNPs and stock solution in P5, line 73-780 as follow.

In this experiment, 100 ppm PtNPs (particle size: 2–19 nm) solution, which was used as a stock solution, were obtained from the manufacturer (Ceramics Craft Co. Ltd. Shizuoka, Japan). The PtNPs were made directly from platinum by irradiation with infrared pulsed laser in liquid according to the Liquid phase laser ablation method. The storage of PtNPs was kept in the dark at room temperature avoiding direct sunlight as the stock solution. Moreover, when using for experiment, the stock solution was diluted in sterile purified water and used each time. Fig.1 shows a transmission electron micrograph of PtNPs used in this experiment.

3. Q: In material and method section "antibacterial testing" add a reference for the protocol utilized.

→ A: We added the reference, which is show in below, in P7 line 111

Ref. 25

Decreased Phototoxic Effects of TiO₂ Nanoparticles in Consortium of Bacterial Isolates from Domestic Waste Water

Ankita Mathur, Jyoti Kumari, Abhinav Parashar, Lavanya T., N. Chandrasekaran, Amitava Mukherjee

Published: October 23, 2015https://doi.org/10.1371/journal.pone.0141301

4. Q: In the discussion section, line no. 243, explain the mechanism of interaction of aerobic and anaerobic bacteria with NPs.

→ A: We added the explanation of the mechanism of interaction of aerobic and anaerobic bacteria with NPs in P15 line 255-P16 line 259.

While, it is inferred that the antibacterial effect of NPs on anaerobic bacteria of oral origin is lower than that on aerobic bacteria. It is considered that this is because the antibacterial property is controlled by the availability of oxygen and the particle size^34)^. These interactions may have shown similar antibacterial activity to *P. gingivalis*, *S. mutans* and *E. faecalis*.

34): Size-dependent antibacterial activities of silver nanoparticles against oral anaerobic pathogenic bacteria. [Lu Z](https://www.ncbi.nlm.nih.gov/pubmed/?term=Lu%20Z%5BAuthor%5D&cauthor=true&cauthor_uid=23440430), [Rong K](https://www.ncbi.nlm.nih.gov/pubmed/?term=Rong%20K%5BAuthor%5D&cauthor=true&cauthor_uid=23440430), [Li J](https://www.ncbi.nlm.nih.gov/pubmed/?term=Li%20J%5BAuthor%5D&cauthor=true&cauthor_uid=23440430), [Yang H](https://www.ncbi.nlm.nih.gov/pubmed/?term=Yang%20H%5BAuthor%5D&cauthor=true&cauthor_uid=23440430), [Chen R](https://www.ncbi.nlm.nih.gov/pubmed/?term=Chen%20R%5BAuthor%5D&cauthor=true&cauthor_uid=23440430). [J Mater Sci Mater Med.](https://www.ncbi.nlm.nih.gov/pubmed/23440430) 2013 Jun;24(6):1465-71. doi: 10.1007/s10856-013-4894-5. Epub 2013 Feb 26.

5. Q: In the discussion section, line no. 268, when LPS reacts with NPs is there any charge based interaction in Gram negative bacteria.

→ A: We added the following in P17 line 283-286.

LPS, which is a component of the cell wall of gram negative bacteria, is negatively charged and has a high affinity to NPs and binds tightly. And it is thought that destruction of LPS arises from LPS and NPs from a physical interaction^37^）.

37）Metal nanoparticles: understanding the mechanisms behind antibacterial activity. Slavin YN, Asnis J, Häfeli UO, Bach H. J Nanobiotechnology. 2017 Oct 3;15(1):65. doi: 10.1186/s12951-017-0308-z.

6. Q: Figure number 3 add an X axis demarcation.

→ A: We add to X axis demarcation in Fig 3 (change to 4).

Reviewer 2

0. Q: As the author pointed out, the NC-PtNPs increase fragmentation and decomposition of albumin, which may limit their practical applications because besides damging the LPs, the proposed NPs can be toxic to host through damaging the albumin.

P19, line 306-307

→ A: Following your suggestions, I added the following sentence.

However, since this study is a limited basic study and a non-specific reaction, further research is necessary for clinical application

1. Q: The authors claim non-colloidal form, but the particle size is 2–19 nm and suspended in water. Comment on it.

→ A: This PtNPs were made directly from platinum by irradiation with infrared pulsed laser. However, we don't have detailed data about non-colloidal or colloidal. Therefore, I would like to revise this paper with the term platinum nanoparticles (PtNPs), rather than using the term non-colloidal platinum nanoparticles (NC-PtNPs).

Thank you for suggesting very important points.

2. Q: Although the NPs are obtained from commercial source, its size should be verified after suspended in water and data should be presented.

P5, line 82-84

→ A: Following your suggestions, we have added an electron micrograph of PtNPs.

3. Q: What will happen to fate of the NPs size and morphology after contacting either albumin or LPS.

P14, line 205-210

→ A: Following your suggestions, we have added an electron micrograph of PtNPs with albumin or LPS.

4. Q: What is mechanism of decomposition?

→ A: Added the following comments regarding the mechanism of disassembly.

P18, line 291-293

Albumin and LPS in contact with PtNPs are thought to be reduced in molecular weight due to cleavage of the protein backbone due to the oxidative degradation reaction or physical interaction caused by the generated ROS^38）^. However, the detailed mechanism is still unknown.

38）Mol Aspects Med. 2014 Feb;35:1-71. doi: 10.1016/j.mam.2012.09.001. Epub 2012 Oct 26.

Protein damage, repair and proteolysis.

Chondrogianni N, Petropoulos I, Grimm S, Georgila K, Catalgol B, Friguet B, Grune T, Gonos ES.
